# High Levels of Oxidative Stress and Skin Microbiome are Critical for Initiation and Development of Chronic Wounds in Diabetic Mice

**DOI:** 10.1038/s41598-019-55644-3

**Published:** 2019-12-17

**Authors:** Jane H. Kim, Benjamin Yang, Amanda Tedesco, Elyson Gavin D. Lebig, Paul M. Ruegger, Karen Xu, James Borneman, Manuela Martins-Green

**Affiliations:** 1 Department of Molecular, Cell and Systems Biology, University of California, Riverside, USA; 2 Department of Microbiology and Plant Pathology, University of California, Riverside, USA; 3 Department of Statistics, University of California, Riverside, USA

**Keywords:** Biofilms, Microbial communities, Chronic inflammation, Experimental models of disease, Bacterial infection

## Abstract

A balanced redox state is critical for proper healing. Although human chronic wounds are characterized by high levels of oxidative stress (OS), whether OS levels are critical for chronic wound development is not known. For these studies, we used our chronic wound model in diabetic mice that has similar characteristics as human chronic wounds, including naturally developed biofilm. We *hypothesize* that OS levels in wound tissues are critical for chronic wound initiation and development. We show that increased OS levels in the wound correlate with increased chronicity. Moreover, without increased OS levels, biofilm taken from chronic wounds and placed in new excision wounds do not create chronic wounds. Similarly, high OS levels in the wound tissue in the absence of the skin microbiome do not lead to chronic wounds. These findings show that both high OS levels and bacteria are needed for chronic wound initiation and development. In conclusion, OS levels in the wound at time of injury are critical for biofilm formation and chronic wound development and may be a good predictor of the degree of wound chronicity. Treating such wounds might be accomplished by managing OS levels with antioxidants combined with manipulation of the skin microbiome after debridement.

## Introduction

Cutaneous wound healing requires a concerted effort of cellular and molecular processes to heal the skin that has been injured^1–3^. It involves four highly regulated overlapping phases: homeostasis, inflammation, granulation tissue formation, and tissue remodeling^1,3–5^. After injury, homeostasis needs to be achieved to prevent blood loss and block the influx of microorganisms from the environment into the blood stream; blood vessels constrict to decrease blood flow and to allow platelets to stick to the injured site. Fibrinogen is activated to form fibrin fibers that associate with platelets and red blood cells to form a clot. Once homeostasis is achieved, blood vessels dilate to allow immune cells, antibodies, nutrients and oxygen to flow into the wound tissue. Molecules, such as growth factors and cytokines released from the platelets during clotting, attract neutrophils to the injury site. These leukocytes secrete proteolytic enzymes and release reactive oxygen species into the wound bed to combat invading bacteria. Monocytes, also recruited to the wound, differentiate into pro-inflammatory macrophages to aid in the inflammatory process by clearing dead neutrophils, cellular debris and bacteria through phagocytosis^5^. They also secrete new growth factors and cytokines that are important for the next phase of healing – proliferative phase.

As inflammation resolves, the proliferative phase begins and rebuilding of new tissue initiates^6,7^. Keratinocytes proliferate and migrate over the wound to create a new epidermis through re-epithelialization. To begin reconstruction of the dermis, fibroblasts and endothelial cells migrate and proliferate beneath the newly formed epithelium, generating new dermal tissue called granulation tissue^2,4,5^. Fibroblasts proliferate and differentiate into myofibroblasts, which aid in healing through contraction of the wound and secretion of extracellular matrix (ECM) proteins such as collagen, a component of the scar tissue. Angiogenesis, a process by which new blood vessels develop by proliferation and migration of endothelial cells, brings nutrients and oxygen to the wound site to support the reconstruction of the granulation tissue^2,5,7^.

Remodeling, which is the last stage of wound healing, begins after re-epithelization and granulation tissue formation. The excess cells are eliminated from the tissue by undergoing apoptosis and being removed by resident macrophages and histiocytes^5^. The tensile strength of the tissue is dependent on various components of the ECM and on fibroblasts in the scar tissue so that the newly formed tissue becomes strong^4^. Finally, the skin barrier is re-established to protect the newly formed tissue from the environment^8^.

Chronic wounds develop when one or more of the processes described above are disrupted or occur out of sequence. Patients with underlying pathological conditions, such as metabolic diseases, are particularly susceptible to developing chronic wounds^9,10^. For example, patients with Type II diabetes have significantly higher probabilities of developing chronic wounds, particularly in the feet, called diabetic foot ulcers (DFUs). Of the ~30 million Americans who have diabetes, approximately 25% will experience a foot ulcer during their lifetime^4,8,9^. DFUs are commonly infected with microbial biofilms that prevent healing since they can be resistant to antimicrobial therapies. When the wound is unable to heal, amputation of the lower limbs may be necessary to contain the spread of infection. DFUs wounds cost the US up to $13 billion a year^11^.

Many chronic wounds persist despite antibiotic therapy, wound debridement, and applications of wound dressings^12,13^. It is known that chronic wounds in humans contain elevated levels of oxidative stress (OS)^5^. OS occurs when an imbalance of redox chemicals exists in the tissues being affected. Reactive species are separated into two major groups, reactive nitrogen species (RNS) and reactive oxygen species (ROS)^14,15^. When oxidizing radicals build up within a tissue, it is usually due to inhibition or insufficiency of detoxifying antioxidant enzymes. Therefore, improper regulation and response to OS results in significant damage to DNA, proteins and lipids^16,17^. Oxidative damage to DNA can lead to oxidized nucleotides and single strand or double-strand breaks. Oxidative damage to amino acid residues can change protein structure, ultimately altering their function in the cell^18^. Given that chronic wounds are known to contain elevated levels of OS^2,19^, providing the wound bed with additional antioxidants has been shown to support healing^20^.

We have previously created chronic wounds in *db/db*^*−/−*^ mice by inhibiting catalase and glutathione peroxidase (GPx), immediately after wounding^20^. These two enzymes are critical antioxidant molecules during wound healing. When 7 mm diameter full thickness cutaneous wounds in *db/db*^*−/−*^ mice are treated with phosphate buffer saline (PBS) at pH 7.4, the wound typically heals around 20–25 days whereas the same size wound made in C57BL/6 mice closes between 11–12 days. Upon treatment with inhibitors for catalase and GPx at time of injury, changes in the wound tissue lead the biofilm-forming bacteria present in the skin to create biofilm within 3–5 days. In our diabetic mouse model, biofilm-forming bacteria are not artificially introduced after wounding; they are present in the skin microbiota prior to wounding and take advantage of the wound microenvironment created by the high levels of OS to form biofilm. 10–15 days after wounding, the amount of the biofilm greatly increases, and the wounds remain open and infected with biofilm for weeks if the mice survive^20^.

Based on these findings, *we hypothesized* that OS is critical for chronic wound initiation and development and that chronicity is directly proportional to the levels of OS in the wound tissue. To test this hypothesis, we treated wounds in *db/db*^*−/−*^ mice at the time of injury with increasing doses of inhibitors to the antioxidant enzymes, catalase and GPx, to create increasing levels of OS in the wound tissue. We show that OS is critical for the initiation of chronicity and that higher the levels of OS induced at wounding, the more severe the chronic wounds become. We also show that, although OS is necessary for development of chronicity, it is not sufficient; the presence of bacteria on the skin is also necessary. We conclude that in order for the wounds to become chronic, we need both increased levels of OS and the bacteria present in the skin.

## Materials and Methods

### Reagents

3-Amino-1, 2, 4-triazole (ATZ) from Tokyo Chemical Industry Co., Ltd. (Portland, OR). Mercaptosuccinic acid (MSA) from Sigma-Aldrich (St. Louis, MO). Buprenorphine (buprenex) from Henry Schein (Dublin, OH). Isoflurane from Henry Schein (Dublin, OH). Tegaderm Film 1624W from 3 M (Maplewood, MN). 10% Povidone-Iodine Solution from Equate (Bentonville, AR). Bovine serum albumin (BSA) from Fisher Scientific (Hampton, NH). Paraformaldehyde (PFA) from Fisher Scientific (Hampton, NH). Goat serum from Sigma-Aldrich (St. Louis, MO). Vectashield from Vector Laboratories, Inc. (Burlingame, CA). Power Block from BioGenex (Fremont, CA). Krystalon Mounting Medium from EMD Millipore Sigma (Burlington, MA).

### Antibodies

The following primary antibodies were used: anti-collagen IV (col IV) ab6586 from Abcam (Cambridge, UK), EGF-like module-containing mucin-like hormone receptor-like 1 (F4/80) MCA497 from Bio-Rad, formally from AbD Secrotech, (Hercules, CA). The following secondary antibodies were used: goat anti-rabbit antibody conjugated with Alexa Fluor 594 A11012, and goat anti-rat antibody conjugated with Fluorescein A10527 from Invitrogen (Carlsbad, CA).

### Chronic wound model

All experiments were completed in accordance and compliance with federal regulations and the University of California policy and procedures approved by the UCR IACUC (Institutional Animal Care and Use Committee). The description of how to obtain chronic wounds in *db/db*^*−/−*^ mice have been published in detail by us previously^20–22^. Briefly, *db/db*^*−/−*^ mice are bred in our conventional vivarium from B6.BKS(D)-*Lepr*^*db*^/J heterozygotes obtained from the Jackson Laboratories (Stock no. 000697). Only *db/db*^*−/−*^ that are 5–6 months old and weigh at least 50 g are used to create chronic wounds. By this age, the mice have had high blood glucose for a long period of time and are fully diabetic and obese, something that does not occur when the mice are 2 months old, the most common age at which db/db^*−/−*^ mice are used for experiments. To remove the hair, the back of each mouse is shaved and Nair applied to expose the skin. To create the chronic wound, one 7 mm full thickness skin excision wound is made under isoflurane anesthesia and then covered with Tegaderm. OS in the wound tissue is increased by using specific inhibitors for catalase and GPx, 3-amino-1, 2, 4-triazole (ATZ) and mercaptosuccinic acid (MSA), respectively. ATZ is injected intraperitoneally at 1 g ATZ/kg of mouse weight in sterile PBS approximately 20 min before surgery. MSA is administered topically onto the wound site under the Tegaderm at 150 mg MSA/kg of mouse weight in sterile PBS within 10 min after surgery. All mice are treated with buprenex, a pain reliever, injected intraperitoneally at 0.05 mg buprenex/kg of mouse weight in sterile PBS before surgery and 6 h after surgery. After wounding, the mice are housed individually in our conventional vivarium to prevent biting and scratching from other mice.

### Creating microbe-depleted wounds

The *db/db*^*−/−*^ mice are normally housed in a conventional vivarium, but for experiments where wounds cannot have bacteria on the skin, we housed the mice separately and away from the normal *db/db*^*−/−*^ colony in special cages supplied with autoclaved water, irradiated bedding and irradiated vivarium chow. The skin of the mouse was wiped and disinfected with iodine and 70% ethanol immediately prior to surgery in order to remove as much of the natural skin microbiota as possible. As soon as the wounds were made, they were sealed with sterile Tegaderm which provides a barrier to external contaminants and environmental bacteria from the cage and bedding. The mice were given the dose of the inhibitors for high levels of OS at wounding.

### Wound area

The area of each wound was measured over time with inSight by eKare Inc. (Fairfax, VA). Briefly, the device was held parallel to the wound and always at the same distance from the wound. The device was calibrated to measure the wound area accurately after manual delineation of the wound margin.

### Histology and histological staining

Wound tissues were collected and fixed in 4% paraformaldehyde (PFA) in PBS for 4–6 h at room temperature. The tissues were washed three times in PBS for 15 min to remove excess PFA; non-crosslinked PFA was quenched by incubation in 0.1 M glycine in PBS for 30 min. The tissues were washed with PBS and then incubated first in 15% sucrose in PBS for 4–6 h at room temperature, and then 30% sucrose in PBS overnight at 4 °C. The tissues were then embedded in optimal cutting temperature compound (OCT), sectioned (8–10 µm thick) in a cryostat, and stained with hematoxylin & eosin (H&E) and Masson’s trichrome (MT) as we have previously described^20^. Staining with picrosirius red (PSR) (Polysciences Inc) was performed as previously described^23^. Briefly, wound tissues embedded in OCT were sectioned and fixed in 4% PFA for 10 min. The sections were rinsed with DI water before stained with PSR (Solution B) for 90 min, rinsed two times with 0.1 N HCl, pH 4.0 (Solution C) for 1 min before rinsing again in DI water. The sections were dehydrated in 70% ethanol for 30 s and allowed to dry before mounting in Krystalon. Stained sections were visualized using a Nikon Microphot-FXA microscope (Nikon Instruments Inc., Melville, NY) and photographed between cross polarizers.

### Immunolabeling

Frozen sections were fixed in 4% PFA in PBS for 20 min. After washing with PBS, excess PFA was quenched with 0.1 M glycine in PBS for 20 min. Tissue sections were incubated in 0.3% Triton X-100 for 30 min when probing for intracellular proteins. Sections were blocked with Power Block for 4 min and then incubated with the primary antibodies in 1% BSA/PBS for 4 h at room temperature, washed three times with 0.1% BSA in PBS and then incubated with secondary antibodies for 1 h at room temperature. After washing with PBS, slides were mounted in Vectashield containing DAPI (4′,6-diamidino-2-phenylindole). Immunofluorescence was visualized and imaged using a Nikon Microphot-FXA fluorescence microscope with a Nikon DS-Fi1 digital camera and NIS-Elements software (Nikon Instruments Inc., Melville, NY).

### Bacterial collection

Bacteria were collected with sterile swabs using the Levine method^24^. Briefly, a sterile swab was rolled around the wound area, approximately 1 cm2 area. Bacterial samples were taken from the skin after injury, and from days 1, 3, 5, 10, 15, and 20 following surgery. For sequencing, the swabs were stored at −80 °C without freezing media.

### DNA extraction

DNA extractions were performed on thawed swabs using the MOBio PowerSoil DNA Isolation Kit (which became the Qiagen PowerSoil DNA Isolation Kit) as described by the manufacturer with a 90 s bead-beating step.

### Bacterial rRNA internal transcribed spacer (ITS) analysis

Illumina bacterial rRNA ITS libraries were constructed as follows: PCRs were performed in an MJ Research PTC-200 thermal cycler (Bio-Rad Inc., Hercules, CA) as 25 µl reactions containing: 50 mM Tris (pH 8.3), bovine serum albumin (BSA) at 500 µg/ml, 2.5 mM MgCl_2_, 250 µM of each deoxynucleotide triphosphate (dNTP), 400 nM of the forward PCR primer, 200 nM of each reverse PCR primer, 2.5 µl of DNA template, and 0.625 U JumpStart Taq DNA polymerase (Sigma-Aldrich, St. Louis, MO). PCR primers targeted a portion of the small-subunit (ITS-1507F, GGTGAAGTCGTAACAAGGTA) and large-subunit (ITS-23SR, GGGTTBCCCCATTCRG) rRNA genes and the hypervariable ITS region^25^, with the reverse primers including a 12 bp barcode and both primers including the sequences needed for Illumina cluster formation; primer binding sites are the reverse and complement of the commonly used small-subunit rRNA gene primer 1492R^26^ and the large-subunit rRNA gene primer 129F^27^. PCR primers were only frozen and thawed once. Thermal cycling parameters were 94 °C for 5 min; 35 cycles of 94 °C for 20 s, 56 °C for 20 s, and 72 °C for 40 s; followed by 72 °C for 10 min. PCR products were purified using a Qiagen QIAquick PCR Purification Kit (Qiagen, Valencia, CA) according to the manufacturer’s instructions.

DNA sequencing (single-end 150 base) was performed using an Illumina MiSeq (Illumina, Inc., San Diego, CA). Clusters were created using template concentrations 2.5 pM and phi X at 107 K/mm2. Data processing was performed with USEARCH v10.0^28^. We used the UPARSE pipeline for de-multiplexing, length trimming, quality filtering and operational taxonomic unit (OTU) picking using default parameters or recommended guidelines that were initially described^29^ and have been updated at https://www.drive5.com/usearch/manual10/uparse_pipeline.html. Briefly, after demultiplexing and using the recommended 1.0 expected error threshold, sequences were trimmed to a uniform length of 149 bp and then dereplicated. Dereplicated sequences were subjected to error-correction (denoised) and chimera filtering to generate zero-radius operational taxonomic units (ZOTUs) using UNOISE3^30^. An OTU table was then generated using the otutab command. ZOTUs having non-bacterial DNA were identified and enumerated by performing a local BLAST search^31^ of their seed sequences against the nucleotide database. ZOTUs were removed if any of their highest scoring BLAST hits contained taxonomic IDs within the rodent family, Fungal or Viridiplantae kingdoms, or PhiX. Taxonomic assignments to bacterial ZOTUs were made by finding the lowest common taxonomic level of the highest BLAST hits excluding unclassified designations. Data were normalized within each sample by dividing the number of reads in each OTU by the total number of reads in that sample.

### Bioinformatics and statistics

One-way ANOVA was used to calculate the significance of the wound area between different doses, followed by Bonferroni’s multiple-comparison test to determine significant differences between groups. One-way ANOVA was also used to calculate the significance of the number of blood vessels and macrophages between different doses, followed by Dunnett’s test to determine significant differences between groups compared to basal level of OS as control. ggplot2 (2.2.1) in R (3.4.0) was used to graph the mean% of the microbiome. Alpha diversity among the treatment groups was analyzed with a statistical model that uses a first-order autoregression covariance structure, assuming heterogenous variances and heterogenous correlations between time and the dose given to each mouse. The least square means were used to perform F-tests and calculate significance.

## Results

### Dose-dependent effect of OS on development of chronic wounds

**Figure 1:**
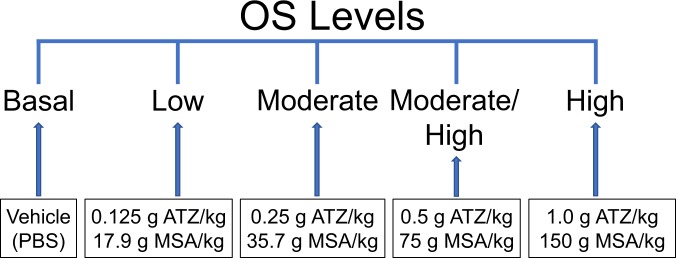
Treatment of the wounds to cause different levels of OS. Different doses of ATZ and MSA, which inhibit catalase and GPx, respectively, were used to induce different levels of OS in the wound. Basal OS levels represents the natural level of OS induced by wound healing in the diabetic mice model *db/db*^*−/−*^.

The *db/db*^*−/−*^ mice have delayed and impaired wound healing but they do not develop chronic wounds. The healing takes approximately twice as long when compared to that of normal C57BL/6J mice. However, when catalase and GPx are inhibited to increase OS to high levels in the wound microenvironment, the wounds become fully chronic within 20 days^20^. These wounds become larger than the original wound because the tissue damaged due to the increased levels of OS disintegrates and contributes to the formation of the biofilm. To determine the importance of the levels of OS in wound chronicity, we performed dose-dependent experiments (Fig. 1). Mouse wounds treated at wounding with only PBS as vehicle are denoted as having *basal level* of OS, representing the natural level of OS induced by the injury. Mouse wounds treated with the inhibitors of catalase and GPx at high doses, 1.0 g ATZ/kg and 150 mg MSA/kg of mouse weight in sterile PBS, are denoted as having *high levels* of OS and go on to develop *fully chronic wounds*. To create wounds with *low levels* of OS, inhibitors at a dose of 0.125 g ATZ/kg and 17.9 mg MSA/kg of mouse weight were administered. For wounds with *moderate levels* of OS, a dose of inhibitors at 0.25 g ATZ/kg and 35.7 mg MSA/kg of mouse weight were administered. For wounds with *moderate/high levels* of OS a dose of inhibitors at 0.5 g ATZ/kg and 75 mg MSA/kg of mouse weight were administered (Fig. 1).

**Figure 2:**
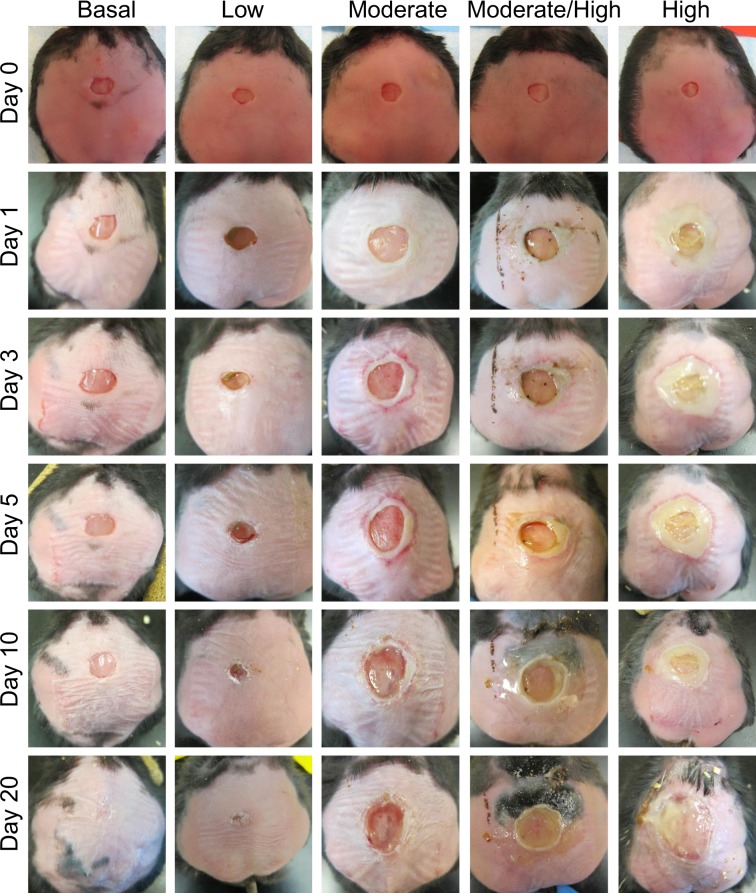
Dose-dependent effects of OS on levels of wound chronicity. Wound healing in *db/db*^*−/−*^ mice is delayed and impaired as OS levels are increased. To create chronic wounds, a full thickness wound is made with a 7 mm biopsy punch and the inhibitors of antioxidant enzyme catalase and GPx applied at the time of wounding. We studied the effects of various levels of OS in the wound tissue: *Basal levels of OS*: Wounds were treated only with vehicle; the wounds were able to close within 20 days and have no biofilm formation. *Low levels of OS*: Wounds were treated with low dose of inhibitors; wound healing is slightly delayed but still occurs normally. *Moderate levels of OS*: Wounds were treated with moderate dose of inhibitors; wound closure is delayed with tissue margins damaged by OS but re-epithelialization occurs and the granulations tissue forms and biofilm can form. *Moderate to high levels of OS*: Wounds were treated with higher doses of inhibitors: Significant damage to the tissue is sustained, wound healing is delayed and wounds are colonized by bacteria that form aggressive biofilm in the wound.

**Figure 3:**
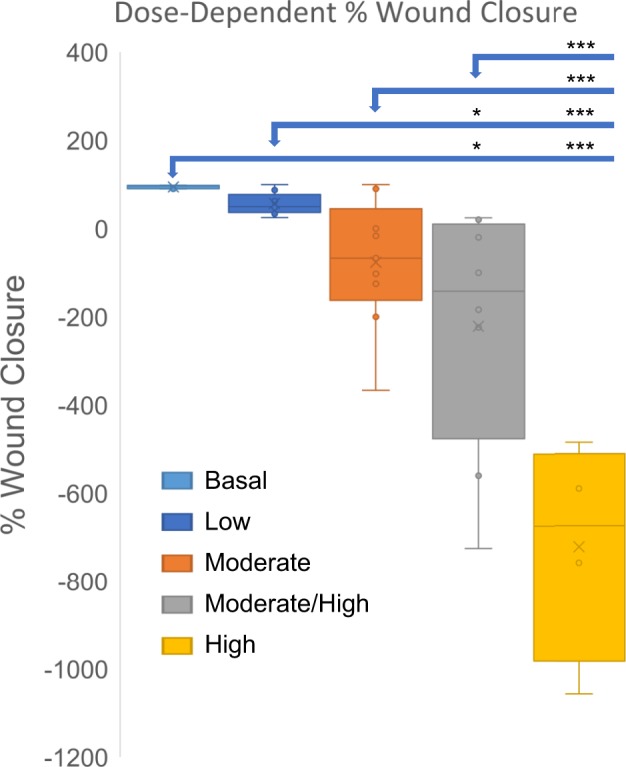
Wound closure is delayed in wounds with higher OS levels. Percent wound closure was calculated by measuring the area at wounding and 20 days after wounding. The wounds with lower levels of OS healed significantly faster than wounds with higher levels of OS. Chronic wounds with the full dose of inhibitors did not heal and had abundant biofilm formation. Wound area results were compared and analyzed using one way ANOVA, followed by Bonferroni’s multiple-comparison test to determine significant differences between groups. *p-value < 0.05, **p-value < 0.01, ***p-value < 0.001. Basal OS, n = 6, low OS, n = 9, moderate OS, n = 8, moderate/high, OS n = 9, high OS, n = 5.

After one 7 mm diameter full thickness wound was made, wounds with basal levels of OS closed in approximately 20 days and those with low levels of OS took a few more days (Fig. 2). Wounds treated with moderate levels of inhibitors showed considerable amounts of tissue damage and took several more days than 20 to close. Increasing the levels of OS further to moderate/high or high levels resulted in extensive damage to the wound tissue with formation of bacterial biofilm. Biofilm formation in wounds with moderate/high OS levels began around days 10–12 days after wounding whereas wounds with high OS levels began much earlier, around days 3–5 after wounding (Fig. 2). Wounds with high OS levels significantly expand after day 10 because the bacteria in the wound break down the dead host skin tissue after it has been damaged by OS. The broken down tissue was used as nutrients for the bacteria and as building material for the biofilm they build in the chronic wounds. Calculation of percent wound closure showed significant differences in area of the wounds between day 0 and day 20 (Fig. 3).

**Figure 4:**
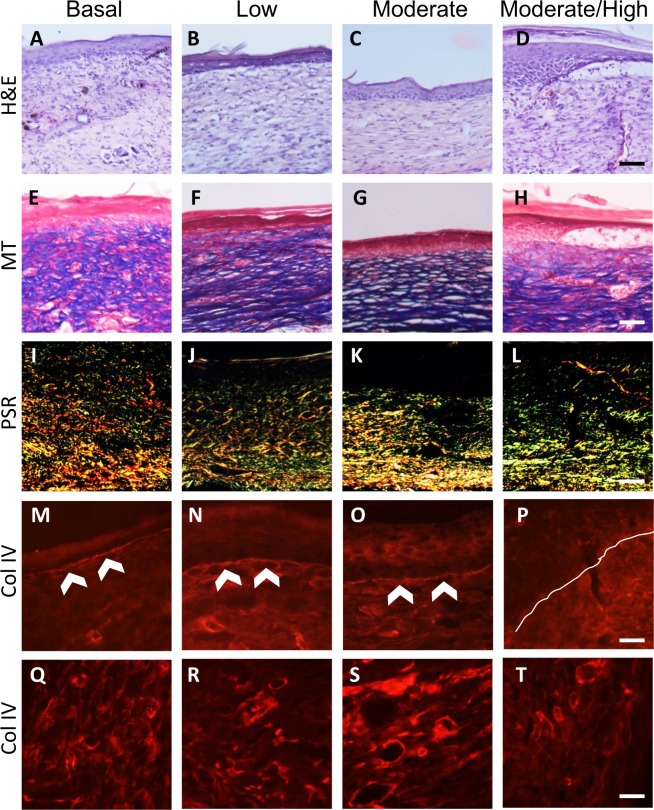
Dose-dependent effects of OS on the quality of wound tissue after wound closure. Cryosections of the skin were taken from wound tissue at the time of complete closure in each basal, low, moderate, and moderate/high levels of OS in the wound tissue. Wounds with high OS levels do not heal so histology was not performed at wound closure. (**A**–**D**) H & E staining of sections of the wound after closure shows formation of an epidermis and quality of the granulation tissue decreasing as levels of OS increased. Scale bar = 100 μm. (**E**–**H**) MT staining of wounds show collagen deposition in the granulation tissue after closure. Scale bar = 100 μm. (**I**–**L**) Picrosirius red staining of wounds show the different composition and location of col I (red) and col III (green) under cross polarizing light. Areas with both col I and col III are shown in orange-yellow. Scale bar = 100 μm. (**M**–**P**) Immunofluorescent staining for col IV shows col IV presence in the basal lamina underneath the epidermis. White arrows indicate the basal lamina formed below the basal cells of the epidermis. The white line in **(P)** A is drawn under where the basal lamina should have formed in wounds with moderate/high levels of OS but is lacking even though the wound has undergone re-epithelialization. Scale bar = 20 μm. (**Q–T**) Images show staining for col IV around the microvessels in the granulation tissue. The density of blood vessels is significantly lower in the wounds with moderate to high level of OS. Scale bar = 20 μm.

To determine how the levels of OS affect wound healing quality, for each treatment, wound tissues were collected after closure (the higher the OS levels the longer the time to closure) and various stains were performed (Fig. 4). H&E staining was performed to visualize overall structure of the wound tissue after closure (Fig. 4A–D). Staining showed that wounds containing basal to moderate levels of OS were able to close with an intact epidermis connected to the newly formed granulation tissue underneath (Fig. 4A–C). The wounds with moderate/high levels of OS were able to form an epidermis; however, the connection between the epidermis and the granulation tissue was poor, indicating poor healing (Fig. 4D). MT staining was used to show whether the wound tissue contained interstitial collagen, which is important for remodeling and maturation of the wound tissue after wound closure (Fig. 4E–H). Compared to wounds with basal levels of OS, the dermis of the wounds with low and moderate levels of OS show more intense collagen staining in the granulation tissue (Fig. 4F–G). However, this could be due to wound compression during sectioning of the sample for histology. Wounds containing moderate/high levels of OS showed that the granulation tissue had a sparse structure and less compact interstitial collagen (Fig. 4H).

The closed wounds were also stained with PSR in order to distinguish between and visualize the distribution of collagen I (col I) and collagen III (col III) in the granulation tissue (Fig. 4I–L). Under cross-polarizing light, the PSR staining showed col I as red and col III as green. When they colocalized, the staining becomes yellowish/orange. In wounds with basal levels of OS, col I appeared as thicker fibers that are evenly distributed in the granulation tissue. Col III fibers were also found distributed in the tissue, with some overlap with col I deeper in the granulation tissue (Fig. 4I). In contrast, wounds with increasing levels of OS showed that after closure, the amount of col I was lower than that of col III and lower than that in the wounds with low OS levels, suggesting that the collagen present in the granulation tissue was primarily col III **(**Fig. 4J,K**)**. This pattern was accentuated in the wounds with moderate/high levels of OS (Fig. 4L).

In order to further examine the quality of the wound tissue with increased OS levels, we immunostained the tissues for col IV which localizes to the basement membrane under the epidermis and in the basement membrane around the microvessels of the granulation tissue. For these studies, we used an antibody specific for col IV (Fig. 4M–T). The basal lamina was well formed under the epidermis of wounds with basal to moderate levels of OS (Fig. 4M–O). However, in the wounds with moderate/high levels of OS, the basement membrane under the epidermis was not well formed (Fig. 4P). The col IV staining in the basement membrane of the blood vessels allowed us to examine the microvasculature in the granulation tissue of the wounds (Fig. 4Q–T). We observed that the blood vessels present in the granulation tissue of wounds with basal, low and moderate OS levels were similar in morphology and abundance (Fig. 4Q–S). The granulation tissue of the moderate/high levels of OS contained significantly fewer blood vessels (Fig. 4T).

**Figure 5:**
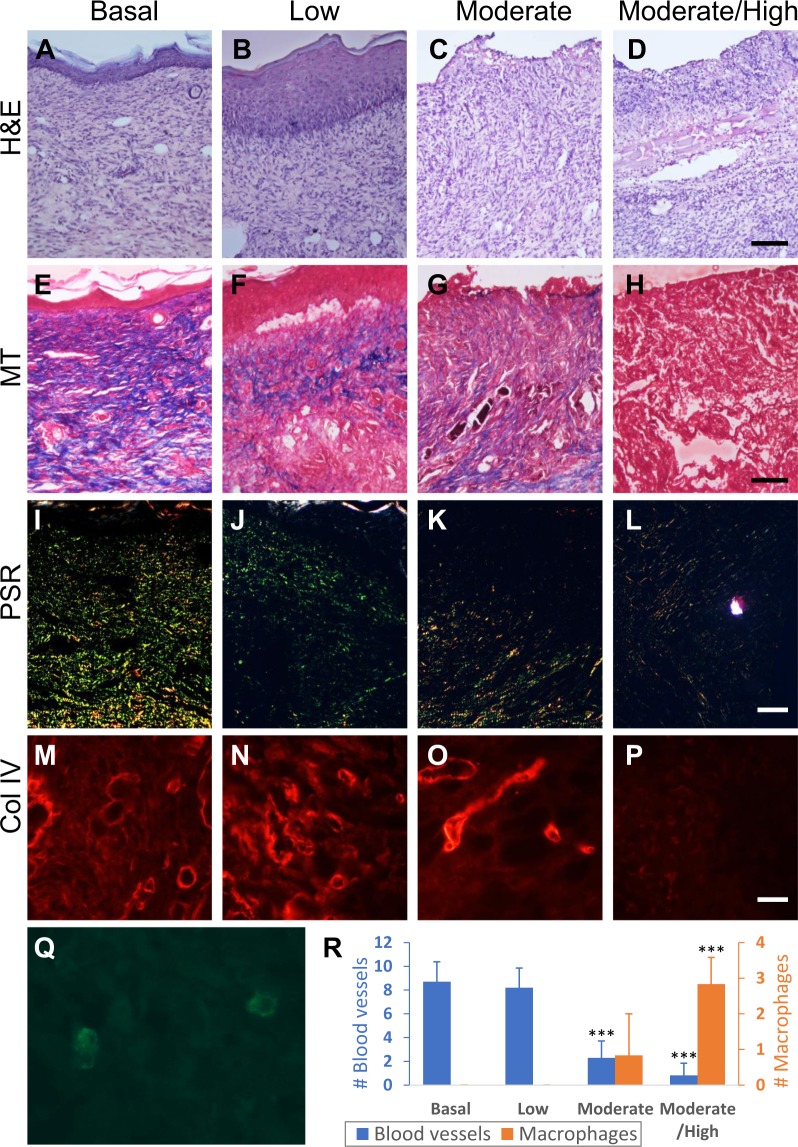
Dose-dependent effects of OS on the quality of wound tissue when the wounds are still opened. Cryosections of the skin were taken from basal, low, moderate, and moderate/high levels of OS wound tissue, 17 days after wounding. (**A**–**D**) H&E staining of sections of the wound at day 17 shows development of an epidermis in wounds with basal and low OS levels. Higher levels of OS in the wound tissues resulted in the wounds being still opened. Scale bar = 100 μm. (**E**–**H**) MT staining of wounds show collagen deposition in the granulation tissue. Dark blue collagen fibers are present in wounds with basal levels of OS. Wounds with low levels of OS showed that the dermal/epidermal junction was weak and the interstitial collagen very sparse. When the wound tissues contained more than moderate levels of OS, interstitial collagen in the wound was absent and the wounds were still opened. Scale bar = 100 μm. (**I**–**L**) Picrosirius red staining of wounds showed col I (red) and col III (green) under cross polarizing light. Areas with both col I and col III are shown in orange-yellow. Wounds with basal OS levels have both col I and col III in the wound tissue whereas wounds with higher OS levels have very little of each col I and col III. Scale bar = 20 μm. (**M**–**P**) Immunofluorescent staining for col IV shows the presence of blood vessels in the granulation tissue. No blood vessels can be found in wounds with moderate/high levels of OS. Scale bar = 20 μm. (**Q**) Staining for F4/80 showed macrophages in the granulation tissue of wounds with higher levels of OS. **(R)** The number of blood vessels and macrophages were counted in 10 and 6 frames (area = 0.02 mm2), respectively, of the granulation tissue. Blood vessel and macrophage results were compared and analyzed to basal level OS wounds using one way ANOVA, followed by Dunnett’s test. *p-value < 0.05, **p-value < 0.01, ***p-value < 0.001.

The data described above showed the quality of wound healing in the presence of increased levels of OS once the wounds have closed. This occurred at different times, with moderate and moderate to high levels of OS taking several days more than wounds with basal and low levels of OS. To observe the effect of OS on the wound tissue structure as the wounds were healing, wound tissues were collected 17 days into the healing process (Fig. 5). H&E staining (Fig. 5A–D) showed that an epidermis has already formed in the wounds with basal and low OS levels (Fig. 5A,B). Wounds with moderate and moderate/high OS levels completely lacked an epidermis, indicating that the wounds were still open at 17 days into healing (Fig. 5C,D). MT staining (Fig. 5E–H) of the wounds with low OS levels showed that collagen deposition was delayed comparing to wounds with basal OS levels at the same time post-wounding (Fig. 5E,F). Both moderate and moderate/high levels of OS resulted in virtually no interstitial collagen deposition at this stage of healing (Fig. 5G,H). The structure of the granulation tissue of wounds with moderate/high levels of OS was also very weak as the tissue was disrupted (Fig. 5H). This pattern was also seen, but to a lesser degree, in wounds with moderate OS levels (Fig. 5G). PSR staining of these wounds showed that as the OS levels increased, the wounds have less deposition of both col I and col III as shown with the MT staining (Fig. 5I–L). Immunofluorescence for col IV showed that the pattern of blood vessels in wounds with basal to low levels of OS (Fig. 5M,N) was similar to that of closed wounds with basal and low levels of OS (Fig. 4Q,R). Wounds with moderate OS levels were open at day 17 but they contained significant numbers of blood vessels (Fig. 5O). In wounds with moderate/high OS levels, the number of new blood vessels in the granulation tissue was low, showing that angiogenesis was delayed (Fig. 5P). Quantitation of the microvessels shows significant decrease as the levels of OS increase (Fig. 5R). Immunostaining for macrophages with F4/80 (Fig. 5Q) to examine the level of inflammation in these wounds showed that, at 17 days into the healing process, the macrophages were no longer present in the wounds with basal, low and moderate levels of OS but were still very much present in the wounds with moderate/high levels of OS (Fig. 5R).

### Wound bacterial microbiome is affected by oxidative stress levels

Because we observed correlation between the levels of OS and the degree of wound chronicity, we investigated whether the levels of OS also affected the degree of biofilm that developed as the wounds became chronic. Analysis of the wound microbiome was conducted by sequencing the bacterial rRNA ITS, a hypervariable region between the 16S and 23S rRNA genes. Variable regions of 16S rRNA gene are more commonly used to obtain descriptions of bacterial communities. However, we used the rRNA ITS region because analysis of this region often enabled identification of bacterial OTUs at species level [25]. From the dose dependent study **(**Fig. 1**)**, 243 bacterial samples were collected on individually wrapped sterile swabs via the Levine method and stored frozen in −80 °C before DNA extraction, library construction and sequencing.

**Figure 6:**
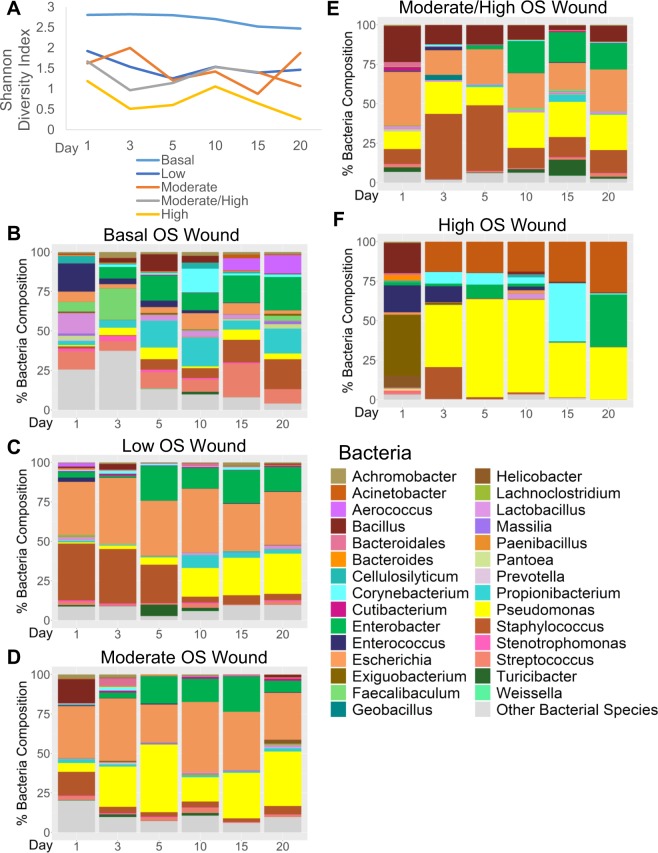
Effects of increasing levels of OS on wound bacteria over time. This analysis was conducted by sequencing the rRNA ITS of DNA isolated from wound swabs. Over 1400 unique OTUs were identified; only the top 30 genus level OTUs were plotted as mean % compositions for each treatment group; the remaining OTUs were combined under “Other Bacterial Species” category. (**A**) Diversity of bacterial microbiome in the wound is affected by OS in a dose dependent manner. Diversity considers species diversity and abundances, not just species richness. Alpha diversity measured with Shannon’s index shows that diversity among doses is significant (p < 0.0001). (**B**) Wounds with basal levels of OS show a diverse bacterial profile throughout wound healing. The same bacterial species are found at almost every timepoint, but their percent composition of the wound is dynamic. (**C**) Wounds with low levels of OS are populated with similar bacteria throughout the profile which includes *E. coli*, *P. aeruginosa, S. xylosus* and *E. cloacae*. (**D**) Wounds with moderate levels of OS have wound profiles similar to wounds with low levels of OS; however, the population of *S. xylosus* is lower and *P. aeruginosa* can be found present in higher proportions earlier in wound development. (**E**) Wounds with moderate/high levels of OS have microbiomes colonized by the same biofilm-forming bacteria as in B and C but with more *S. xylosus* present in the earlier times. (**F**) Wounds with high level of OS can be dominated by more aggressive biofilm-forming bacteria, such as *P. aeruginosa* and *A. johnsonii*. Their colonization begins early and quickly expand their population to outcompete other bacteria in the wound. Basal OS, n = 6, low OS, n = 9, moderate OS, n = 8, moderate/high, OS n = 9, high OS, n = 5.

To determine whether a correlation between the diversity of bacteria and wound healing outcomes existed in our chronic wound model, we calculated the alpha diversity of bacteria in the wounds using the Shannon diversity index and performed statistics accounting for repeated measures (Fig. 6A). Shannon diversity index is a measure of both species richness (number of taxa) and evenness (a measure of the relative abundances) of each of the species. Low indices indicated lower diversity, found typically in infections (e.g. one microorganism dominates and causes disease). High indices indicated higher diversity, found typically in stable, healthy communities. In the following analysis we discovered that the level of OS significantly contributed to a difference in Shannon diversity (p-value < 0.0001). The diversity across time was also significant (p-value < 0.0198). The greatest difference in diversity is between wounds with basal levels of OS and wounds with low, moderate, moderate/high, and high levels of OS (p-values = 0.0007, = 0.0006, < 0.0001, and < 0.0001, respectively). Wounds with closer levels of OS had similar diversity in their bacteria: low and moderate levels (p-value = 0.9996), moderate and moderate/high (p-value = 0.9324), and moderate/high and high (p-value = 0.2455). Wounds with low and moderate OS levels had a Shannon diversity index that was very different from wounds with high OS levels (p-value = 0.0523 and 0.0860, respectively).

Wounds with basal levels of OS had a bacterial microbiome profile that was dynamic and diverse. Diversity was maintained as the wound healed. Several species were present in the wound throughout healing, with the percent composition changing in the wound over time. The dynamics of the microbiome kept the diversity high throughout the healing (Fig. 6B). Interestingly, the presence of biofilm-forming bacteria such as *Enterobacter cloacae*, *Streptococcus thermophilus*, *Propionibacterium acnes*, *Staphylococcus xylosus*, *Pseudomonas aeruginosa*, *Corynebacterium sp*., *Acinetobacter johnsonii* and, *Achromobacter xylosoxidans*, was observed in these wounds but never with biofilm formation. Wounds treated to create low levels of OS were populated with a number of bacterial species found in the wounds with basal OS levels but in different proportions; only a few bacteria dominated the wound: *E. coli*, *E. cloacae*, *S xylosus* and *P. aeruginosa* (Fig. 6C). In days 1–3, a majority of the wound was colonized by *E. coli* and *S. xylosus*. At day 5, *E. cloacae* and *P. aeruginosa* began to colonize the wound until day 10 when *S. xylosus* appeared to be outcompeted by these two bacterial species. The relative abundances among *E. cloacae*, *E. coli*, and *P. aeruginosa* were stabilized by day 10 and there was no biofilm formation; the wound healed similarly to basal OS level wounds (Fig. 2). This observation showed that these bacteria do not form biofilm and colonize the wound in a pathogenic manner unless the levels of OS reached a specific threshold (Fig. 6B). In contrast, wounds with moderate levels of OS, which had a similar profile as wounds with low levels of OS, contained some biofilm (Fig. 6D). With the presence of damaged tissue caused by the MSA, the microenvironment began to provide conditions that are conducive to biofilm development. In these wounds, *S. xylosus* did not comprise a large percentage of the microbiome; its presence was observed on day 1 at 15% of the population and in the subsequent days less than 5%. *P. aeruginosa*, a known biofilm-forming species of human chronic wounds, very quickly colonized the wound after wound initiation; at day 3, it composed more than 25% of the wound, increasing to more than 40% by day 5. Other bacteria such as *E. coli* and *E. cloacae* competed with *P. aeruginosa* for dominance in these wounds. Wounds treated with moderate/high levels of OS in the wound were colonized by the same biofilm-forming bacteria found in the wounds with low and moderate levels of OS (Fig. 6E). Wounds with high levels of OS were dominated by the most aggressive biofilm forming bacteria such as *P. aeruginosa* and *A. johnsonii* which began their colonization at 24 hours after wounding and quickly expanded their populations, outcompeted all other bacteria in the wound and formed strong biofilm (Fig. 6F).

While the average percentage of bacteria in the wound showed multiple bacteria present in a wound in a single day, individual mice with high levels of OS typically showed a single or a couple of bacteria dominating the wound over the course of several days. For example, a mouse wound containing high level of OS had a microbiome entirely composed of *P. aeruginosa* starting from day 3, while another was entirely composed of *A. johnsonii* (data not shown). We observed that when wounds contain low levels of OS, the diversity of bacteria during healing was high. Contrarily, when wounds contain higher levels of OS, the wounds were open and have lower bacterial diversity. These wounds were ultimately colonized by a few or a single bacterial species.

### Oxidative Stress and the microbiome are each necessary but not sufficient for chronic wound initiation

**Figure 7:**
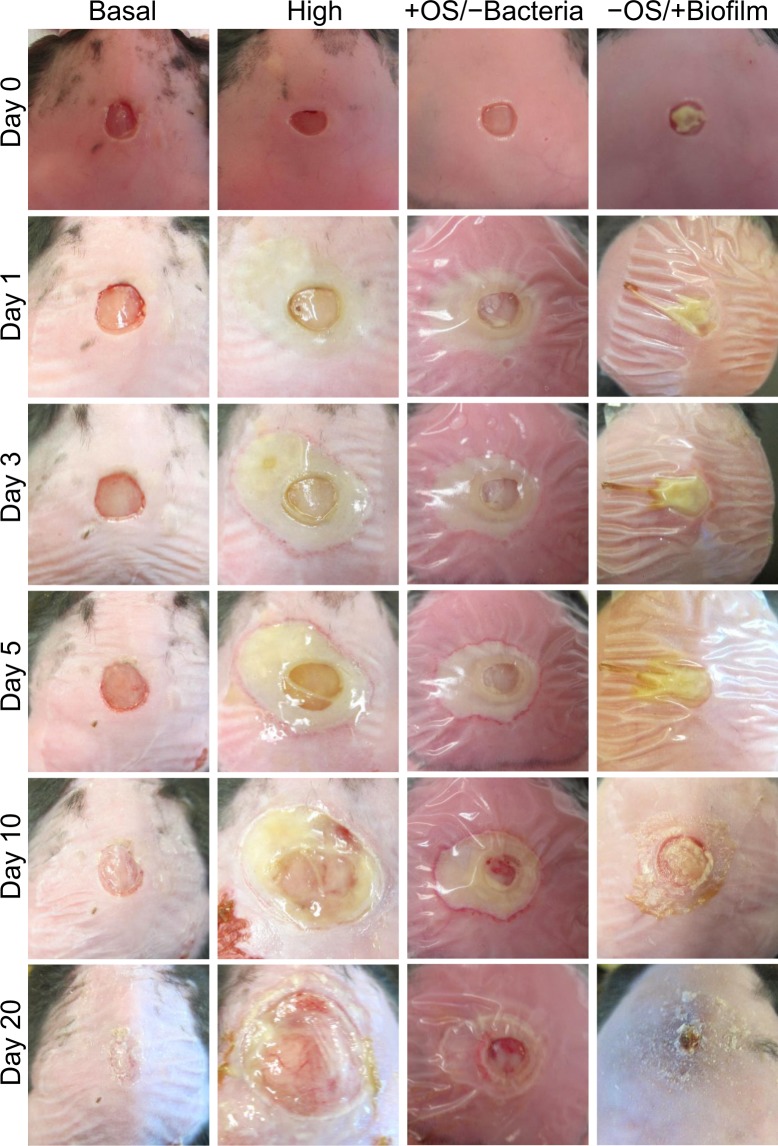
Both high levels of OS and biofilm-forming bacteria are needed to create chronic wounds. To test whether OS and bacteria are critical for chronic wound formation, wounds were either cleaned to remove skin microbiome or transplanted with chronic wound biofilm in the presence or absence of OS. *Basal levels of OS*: wounds heal within 20 days and don’t form biofilm. *High levels of OS*: wounds develop into chronic wounds with biofilm formation. +*OS/−Bacteria*: When high levels of OS were induced with skin microbiome removed, the tissue sustained damage from OS but the wound did not develop into a chronic wound. *−OS*/+*Biofilm*: When wound tissues were transplanted with mature biofilm from other chronic wounds in the absence of additional OS, the biofilm breaks down and the wound heals much like wounds with basal OS levels.

The wound profiles showed that OS had a significant impact on wound healing initiation development and progression. We determined whether OS alone was necessary and sufficient to create chronic wounds without the presence of bacteria in the wound. For that, we removed the bacteria from the skin before wounding and then created high levels of OS in the wound tissue by inhibiting catalase and GPx at wounding (Fig. 7). Compared to wounds with high levels of OS, in which the bacteria were not removed and go on to become chronic, the wounds in the absence of bacteria but with high levels of OS, did not become chronic. Cell death by necrosis in the tissue around the wound was clearly visible but biofilm did not develop and the wounds did not become chronic; bacterial infection and biofilm were not seen at 20 days post-wounding when the wounds would normally become fully chronic in the presence of both high levels of OS and bacteria **(**Fig. 7, column 3).

**Figure 8:**
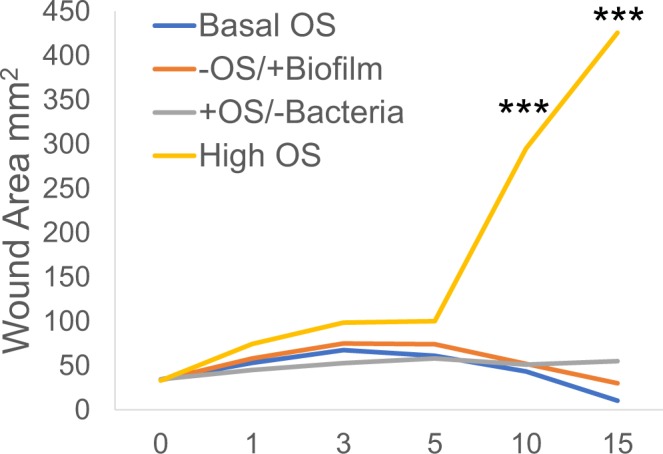
Wound healing is delayed in wounds with OS and bacteria. Measurements of the wound area shows that the area of wound with either OS or biofilm do not increase significantly like wounds with high levels of OS. Wound area results were compared and analyzed using one way ANOVA, followed by Dunnett’s test to determine significant differences between wound areas to basal OS levels. *p-value < 0.05, **p-value < 0.01, ***p-value < 0.001. Basal OS, n = 5, +OS/−Bacteria, n = 4, −OS/+Biofilm, n = 7, high OS, n = 4.

To determine whether bacteria alone were able to make biofilm in the wounds without an environment with high levels of OS, we transplanted biofilm from fully chronic wounds to new wounds without increasing the OS levels in the wound (Fig. 7, column 4). These wounds did not become chronic and went on to heal, much like non-chronic wounds, despite the presence of biofilm obtained from other chronic wounds (Fig. 7, column 4). Wound areas were determined in all four conditions. We showed that wounds with high OS levels had an area significantly increased after day 10 when compared to the areas of the other groups (Fig. 8). These results show that OS and the microbiome in the skin were each necessary but not sufficient to achieve chronicity.

## Discussion

OS has many important roles in regulation of wound healing^5,32^. At basal levels, it creates an environment conducive to initiation of the healing process involving all four phases of healing that we described in the introduction. Increasing OS after wounding will derail these processes. Excessive levels of ROS and RNS, which are highly reactive, can damage DNA, proteins, metabolites and lipids in the wound. Damage sustained by DNA in the form of oxidized nucleotides or single/double stranded breaks initiates various response to DNA damage pathways in order to repair oxidative damages and also regulates fundamental cell cycle processes. However, if the damage cannot be corrected or reversed, this signals to cells and tissues to undergo unregulated cell death in the form of necrosis. Damaged metabolites or oxidized lipids can disturb the cellular redox balance and lead to mitochondrial dysfunction, which can also lead to cell death. Inflammation, whose initiation and termination are strictly controlled in order to facilitate healing, is highly sensitive to the redox state of the wound and can lead to a stronger inflammatory response or prolong the process beyond normal regulation. Damage to regulatory chemicals such as chemokines, cytokines, and growth factors will prevent proper regulation of leukocyte chemotaxis and function in response to injury. Regulation of angiogenesis (microvessel development) is also disturbed by high levels of hydrogen peroxide and nitric oxide leading to inhibition of microvessel development.

**Figure 9:**
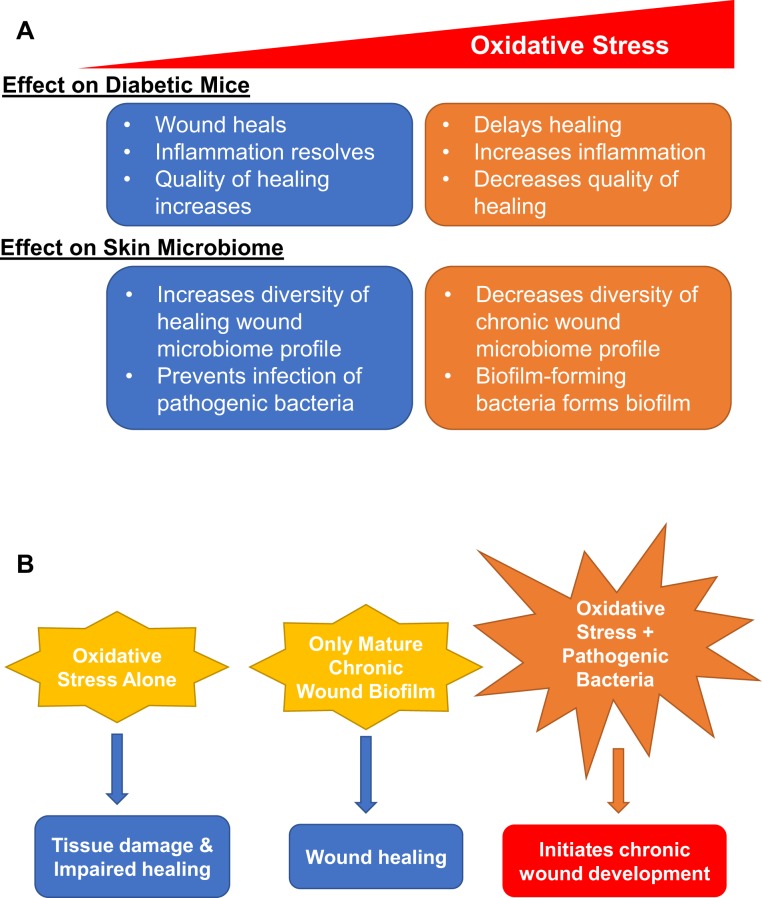
Schematic summary of the effects of OS on wound healing and the bacterial microbiome. (**A**) OS has a significant effect on both the diabetic mouse and the skin bacteria that lead to the development of chronic wounds in the mouse in a dose dependent manner. (**B**) OS and chronic wound biofilm alone are not sufficient to create chronic wounds. Only when both OS and skin bacteria are present does a chronic wound development.

The studies presented here showed that the development of chronic wounds in diabetic and obese mice was directly proportional to the levels of OS in the wound tissue (Fig. 9A). When wounds were treated with inhibitors of catalase and GPx to increase OS levels in the wound, wounds treated to cause low levels of OS closed just slightly delayed when compared with wounds with basal levels of OS (*db/db*^*−/−*^ non-chronic wounds). The quality of healing and the % of bacteria present in the wounds were similar to non-chronic wounds and neither form biofilm. Wounds containing moderate levels of OS were further delayed in closure; they remained open for a longer period of time but the wound underwent re-epithelialization. The basal lamina under the epidermal cells was also developed and the quality of wound healing after wound closure was similar to that of wounds with low levels of OS. Wounds with moderate/high to high levels of OS had a higher degree of tissue disorganization, had considerably larger area compared to the original wound and developed strong biofilm around 10 days after injury. Wounds with high levels of OS did not close and began to develop biofilm with 2–3 days after wounding. The importance of levels of OS is emphasized when observing its effect on the bacteria that colonized the wound. Wounds with lower levels of OS generally showed more dynamic shifts in population percentages over time, whereas wounds with higher levels of OS showed decreased dynamic shifts with fewer biofilm-forming bacterial species colonizing the wounds. Therefore, the levels of OS were important in the microbiome that developed in the wound; increased OS levels decreased the diversity of the wound microbiome by providing a microenvironment conducive to biofilm-forming bacteria to thrive and form biofilm in the wound. Therefore, the levels of OS in a wound at the time of injury are critical for biofilm formation and chronic wound development and may be a good predictor of the degree of wound chronicity. We also found that, when mature bacterial biofilm was transplanted onto wounds after injury without increasing OS levels, wounds were able to heal in a timely manner. Similarly, wounds n/a treated to eradicate existing skin microbiome and administered inhibitors for antioxidant enzymes to increase OS levels, underwent moderate tissue damage without the development of a chronic wound (Fig. 9B).

We also show that the quality of healing after closure of wounds treated with moderate levels of OS can be similar to wounds with low or basal levels of OS and they do not develop biofilm. However, in some cases wound healing is delayed with biofilm formation that is not as strong as that found in wounds with moderate/high OS levels. Because damage to the tissue is not extensive, this results in the formation of mild chronic wounds. These results suggest that in the wounds with moderate levels of OS, other factors such as the types of bacteria present in the skin of the mouse, have strong effects on the healing outcome. Circumstances, such as bacterial interactions in the wound, may prevent strong biofilm-forming bacteria from colonizing the wound and forming biofilm. If this is the case, wound healing will not be very delayed and the wound closes even if the levels of OS in the wound are moderate. However, if a strong biofilm-forming bacteria is abundant in the skin to start with, moderate levels of OS might create a microenvironment conducive for biofilm formation preventing healing.

Wounds with moderate to high levels of OS are able to undergo re-epithelialization, but the connection between the epidermis and the granulation tissue underneath is weak. The basement membrane lacks col IV, indicating that the wounds have no basal lamina. These wounds also had a higher number of macrophages, indicating that inflammation has continued days past when inflammation in healing wounds should have resolved. We conclude that wounds with excessive levels of OS are at increased risk of having impaired healing, biofilm formation and developing chronic wound. Studies to determine whether inducing high levels of OS as the healing is progressing results in derailing of the healing process and causes chronicity will be under way soon.

In humans, the skin microbiome has important implications in cutaneous wound healing^32–34^. Culture-independent methods using 16S rRNA and/or shotgun metagenomic sequencing, surveyed complex wound microbiomes across domains with dynamic interactions between microbes over time corresponding to healing and impaired wounds^33,35–37^. Host-microbe interactions studies in skin further stressed the complex relationship between wound healing processes and the skin-residing microbes^38,39^. Of the bacteria found in human chronic wounds, the following were found in our chronic wound model: *Acinetobacter sp*.^40–44^, *Bacillus sp*.^42,43,45,46^, *Enterobacter sp*.^40,41,43,44,47^, *Escherichia sp*.^40,41,43,44,46,48^, *Propionibacterium sp*.^45,46^, *Enterococcus sp*.^40,42,43,45,47,48^, *Pseudomonas sp*.^40–42,45–48^, and *Staphylococcus sp*.^40,43,45–47^. In our *db/db*^*−/−*^ mice, much like in humans, the bacterial profile can diverge from litter to litter and even in cage mates so that each mouse followed over time shows an individualized profile in the % of the bacterial species colonizing the respective wound in response to OS levels. These finding indicate that our mouse has the ability to form mature biofilm in the chronic wounds from the skin microbiome that parallels that of human chronic wounds.

High OS in human chronic wounds can be a result of a hypoxic wound microenvironment or ischemia, which significantly impairs wound healing in human wounds^49^. When tissues have poor vascularization and blood flow, the wound tissue fails to have sufficient oxygen pressure and nutrients to support healing and instead lead to the breakdown of the tissue or development of ulcers and infection. This effect is worsened especially in lower extremities of diabetic patients because they also suffer from chronic hyperglycemia, neuropathy, arterial insufficiency, and dysfunctional leukocytes. Angiogenesis, which we observed to be delayed in wounds with high OS levels, is also found to be improperly regulated and delayed in human chronic wounds. We observed that re-epithelization is delayed as wounds were subjected to higher levels of OS. Human chronic wounds are also difficult to heal because re-epithelialization of the wound is either delayed or does not occur. Histology of human chronic wounds shows that at the wound edge, the epidermis is thick and hyperproliferative, the epithelial tongue does not form and epithelial migration does not occur. This suggests that important keratinocyte functions, such as activation, differentiation and migration, are aberrant or disturbed^50^.

***In conclusion***, the degree of chronicity of the wounds depends on the levels of OS when the wound is first created. Wounds with lower levels of OS heal and are colonized by a diverse bacterial community. These wounds may have biofilm-forming bacteria present in the wound, but these bacteria do not form biofilm. Wounds with higher levels of OS are colonized by pathogenic bacterial species that form biofilm and do not heal normally. Furthermore, the levels of OS present in the wound after injury greatly impacts wound healing progression and prognosis and also it affects the type of bacteria that colonize the wound, acquiring either pathogenic or commensal/non-pathogenic roles. Both OS and bacteria in the skin are crucial for the development of chronic wounds; high levels of OS are necessary but not sufficient for chronic wound development, and mature bacterial biofilm in the absence of high OS levels does not lead to development of chronic wounds. These studies provide a platform to further study the effects of OS in both the progression of wound healing and the dynamics of the microbiome over time, potentially leading to new strategies to treat these recalcitrant wounds.
